# Progress of Medicine in the Western States

**Published:** 1881-04

**Authors:** 


*Collaborators:*

DR. L. W. CASE, DR. R. TILLEY,
DR. BYRON W. GRIFFIN, DR. W. L. DORLAND,
DR. H D. VALIN.

### PRACTICAL MEDICINE.

### PROGRESS OF MEDICINE IN THE WESTERN STATES.

Dr. R. W. Erwin, Bay City, Michigan, gives an interesting account of a rhino-plastic operation performed by him in 1879. The new nose thus formed looks much like the natural organ, at least in the cut, and an interesting fact is that the smell, absent for years, was also restored.

The points of most interest derived from the case are:

" *First.* The entire structure should come from the forehead without regard to hair.

" *Second.* It should be large enough, especially in the vertical diameter, to admit of leaving the pedicle for several months undivided. The column for the septum must be long, else it will continue to draw down the tip. The leaving the pedicle attached does more than anything else to prevent the organ from becoming flat. While left it will be necessary to use a little pressure over it to prevent retraction. Court plaster will do, and may be applied by the patient himself. Should the patient be unwilling to exercise this care it would be better to divide the pedicle as customary.

" *Third.* Long after the healing, a compress on each side of the nose should be worn part of the time (nights will do) to insure symmetry and permanency of shape.

"*Fourth.* No skin should be wasted in preparing the parts for the reception of the flap, no matter how small. It should be dissected up, and turned over to form a partial cover for the exposed raw surface beneath the bridge."—*N. Y. Med. Record*, Feb. 12, 1881.

